# Cumulative advantages and social capabilities in scientific mobility in the Health Sciences: The Spanish case

**DOI:** 10.1371/journal.pone.0173204

**Published:** 2017-03-15

**Authors:** Pedro Aceituno-Aceituno, Lorenzo Melchor, Joaquín Danvila-del-Valle, Carlos Bousoño-Calzón

**Affiliations:** 1 Departamento de Administración y Dirección de Empresas y Economía, Universidad a Distancia de Madrid (UDIMA), Collado Villalba (Madrid), España; 2 Independent Researcher, London, United Kingdom; 3 Departamento de Administración y Dirección de Empresas y Economía, Universidad a Distancia de Madrid (UDIMA), Collado Villalba (Madrid), España; 4 Departamento de Teoría de la Señal y Comunicaciones, Universidad Carlos III de Madrid (UCIIIM), Leganés (Madrid), España; IUMPA - Universitat Politecnica de Valencia, SPAIN

## Abstract

**Background:**

The big problem in global public health, arising from the international migration of physicians from less-developed to more-developed countries, increases if this migration also affects scientists dedicated to health areas. This article analyzes critical variables in the processes of Spanish scientific mobility in Health Sciences to articulate effective management policies for the benefit of national public health services and the balance between local and global science.

**Methods:**

This study develops a survey to measure and analyze the following crucial variables: research career, training, funding, working with a world-class team, institutional prestige, wages, facilities/infrastructure, working conditions in the organization of the destination country, fringe benefits in the organization of the destination country and social responsibility in the organization of the departure country. A total of 811 researchers have participated in the survey, of which 293 were from the health sector: Spanish scientists abroad (114), scientists that have returned to Spain (32) and young researchers in Spain (147).

**Results:**

The most crucial variables for Spanish scientists and young researchers in Spain in Health Sciences moving abroad are the cumulative advantages (research career, training, funding and institutional prestige) plus wages. On the other hand, the return of Spanish scientists in the Health Sciences is influenced by cumulative variables (working with a world-class team, research career and institutional prestige) and also by other variables related to social factors, such as working conditions and fringe benefits in the destination country. Permanent positions are rare for these groups and their decisions regarding mobility depend to a large extent on job opportunities.

**Conclusions:**

Spanish health organizations can influence researchers to return, since these decisions mainly depend on job opportunities. These organizations can complement the cumulative advantages offered by the wealthier countries with the intensification of social factors.

## Introduction

Accelerated international migration, from less developed to more developed countries, has reduced the number of physicians in poor countries [1]. This one-sided migration is a *big global public health problem* that also affects middle-income countries [2]. The incorporation of scientists to health services not only encourages medical research in health services [3,4], but also improves researchers´ performance in translational research [5]. However, when the *brain drain* involves health scientists, these beneficial effects, as well as the rapid incorporation of research advances into clinical practice, do not occur. Curing patients becomes a long and expensive process and results in a worse diagnosis and treatment [6].

The business sector also benefits from inventing, testing and commercializing new drugs, vaccines, and medical equipment that are normally protected by patents and may provide better diagnoses and treatments for patients. Government documents often take into account investigation results [7]. Therefore, medical research makes an important contribution both to social aspects, such as improving the quality and expectancy of life, and to economic aspects, such as wealth and employment creation [8].

Experts on scientific mobility prefer to speak of “brain circulation” rather than “brain drain” or “brain gain”. According to this *circulation*, scientists travel through different countries doing their work and all benefit from mutual collaboration. However, this vision of a world where scientists are moving to and fro is far from the reality. Scientists continue to be attracted by countries that have more resources [9–11] and offer better wages [12].

International collaboration is a national research enrichment factor, and benefits both researchers that collaborate in their country of origin [13] and, especially, those working abroad [14–16]. However, this mobility has its limits (personal and family reasons, excessive trips, for example) and may be biased towards the interests of the largest countries. Therefore, the challenge is to find a balance between local and global science [17].

In pursuing this balance, it is worth noting that governments’ influence in order to get scientists repatriated is quite limited [18] and that the reasons for them returning are mainly personal or family reasons. Market conditions in low-scientific-mobility countries may also have an influence on scientists in their decision to return; although it is not easy to assess due to the difficultly in tracking these researchers abroad [19]. The scarcity of data for this group is common to other studies such as Baruffaldi and Landoni’s [20].

Considering the labor market conditions, it is important to highlight *the cumulative advantages* concept as coined by Merton [21] as an enabler for young researchers to improve their competitiveness in the future. These cumulative advantages are related to trained capacity, structural location, available resources, institutional prestige and an optimal working environment for world-class team [22]. Following this contribution by Merton [21, 22], later research has highlighted the importance of the scientists’ research careers in order to explain their scientific performance [23], especially in their early stages [24]. Merton [21] has referred to this concept as the “The Matthew Effect in Science” as inspired by the biblical passage: “For unto everyone that hath shall be given, and he shall have abundance: but from him that hath not shall be taken away even that which he hath” [25,26].To capitalize on these cumulative advantages, scientists are extremely competitive and leave their countries in order to achieve their full scientific potential, otherwise they would stay [27].

Speaking of *brain drain*, the loss of knowledge is explained by the scientists’ non-return. However when considering the *brain circulation* approach, this loss can also be tracked down to the removal or weakening of ties with the country of origin [28]. Both to prevent this from happening and to allow the countries of origin to access their researchers’ knowledge and professional networks [29], it is important to keep a close collaboration with national researchers abroad [30, 31]. This is particularly relevant for their home institutions [32], since those are the places to which these scientists typically return [33].

The Spanish Health Sciences case suggests the need to improve some of the cumulative advantages as discussed above. Examples of areas which could be improved in the Spanish system are the lack of recognition, deficiencies in structuring research careers [34–38] as well as insufficient research funds since the 2008 economic crisis [39]. Also, the most recent *National Plans for Research*, *Development and Innovation* (R&D&i) (2004–2007 [40, 41], 2008–2011 [42] and 2013–2016 [43]) refer to both these cumulative advantages and those related to the improvement of training. In the same vein, the latest *Spanish National Plan for R&D&i 2013–2016* [43] highlights the relevance of the scientific reputation of the institutions. To foster their reputation, the *Sistema Español de Ciencia Tecnología e Innovación* (SECTI) has established an *Acción Estratégica de Salud* (AES) to support their collaboration in Scientific and Technological Networks for excellence.

Data concerning the scientists associated with *the Sistema de Comunicación con Investigadores en el Exterior* (RedIEX) *of the Spanish Foundation for Science and Technology/Fundación Española para la Ciencia y la Tecnología* (FECYT), 44% of which belonged to the Health Science in 2010, support the above assessment. The key factors to note were the expectations to continue their scientific career improvement and the possibility of a better remuneration [44]. Additionally, the most recent studies carried out along with the main scientific Spanish societies abroad (*Society of Spanish Researchers in the United Kingdom/Comunidad de Científicos Españoles en el Reino Unido*-SRKU/CERU, *Científicos Españoles en la República Federal de Alemania*-CERFA, *Association of Spanish Scientists in Sweden/Asociación de Científicos Españoles en Suecia*-ACES y *Comunidad de Españoles Científicos en Estados Unidos*-ECUSA) show the most important reasons for Spanish scientists in the Health Areas to leave Spain to be firstly research career progress (72%) and secondly, the lack of employment opportunities (45.2%). In this case, most scientists consider the lack of funding (90.4%) and the lack of employment opportunities (89.4%) as their main obstacles to returning [45]. Finally, it is worth mentioning that Spanish scientists exhibit higher levels of collaboration with international institutions than with national or home institutions [46].

Our contribution to this ongoing debate is to provide additional data and to analyze key variables for the Spanish scientific mobility in the Health Sciences. Our results may also be of interest for countries in similar conditions. In summary, more effective management policies may improve not only National Public Health Services, but also help to achieve a better balance between local and global science.

## Materials and methods

This study includes three groups of Spanish scientists: 1) those working abroad (SSA), 2) those that have already returned to Spain after working abroad for at least one year (SRS), and 3) the Young researchers in Spain (YRS) group which was also added because it is highly susceptible to going abroad to find cumulative advantages [21, 22]. This latter group of researchers is defined as those Spanish scientists in Spain below 41 years old. Our survey was online from December 2014 to April 2015.

### Ethics statement

This study has been approved by the *Universidad a Distancia de Madrid (UDIMA) Ethics Committee*. The completion of its questionnaire was on an anonymous and voluntary basis. All participants provided their informed consent to participate in the study, as embedded in the questionnaire. The authors of this study did not interact with the participants in any way. Data are displayed in the tables and supporting information provided with this paper. All the information supporting this study is available for interested researchers.

### Participants and scope

Recent *National Plans for R&D&I* (*2004–2007* [40, 41], *2008–2011* [42] and *2013–2016* [43]) have provided public financial support for the national and international mobility of researchers in all areas of knowledge, as given in the *Sistema Español de Ciencia y Tecnología* (SECTI). Additionally, Spain has supported *Marie Skłodowska-Curie* actions within the *European Horizon 2020 Framework Programme* to foster scientists’ training and mobility in all the knowledge areas. These actions jointly with those by the *European Research Council* (ERC) are fundamental to increasing the European base of highly qualified scientists. Finally, co-funding schemes, open to other member countries, are set up in National and Regional Human Resources policies following the latest *Framework Programme ‘COFUND’* guidelines [47]. Therefore, comparing the Health Sciences against other knowledge fields can assess the variables associated with good practice, and these results applied to improve the scientific mobility in the Health Sciences.

As previously mentioned, tracking researchers abroad is a difficult task [19]. In the case of the SSA group, although there have been several attempts to carry out a census, there is no official data on the number of researchers in this group. Thus, although Antoni Valero-Cabré, a Spanish academic, collected up to 2100 names in 2003 [48], Salvador Ordoñez, Secretary of State for the *Ministerio de Educación y Ciencia* (MEC), stated in an interview that MEC had registered just over 1000 scientists in 2005 [49]. Years later, in 2010 [43], the *Spanish Foundation for Science and Tecnology/Fundación Española para la Ciencia y la Tecnología* (FECYT) had census data of around 1300 researchers. In 2013, members of Spanish scientific associations working abroad added up to a population over 2000 researchers [50]. In the case of the SRS group, there are no official data either, although the *Ministerio de Ciencia e Innovación*, claimed that 716 national scientists had returned to Spain thanks to the *Ramón y Cajal Fellowship Program* [51] in 2008. The situation for the YRS group is similar to the other groups.

In summary, there is a lack of available data on the population and profile of these researchers. This work alleviates this problem by collecting data throughout different associations and organizations that support the Spanish scientific careers development. In the case of the SSA group, data are compiled by using the lists of the following associations: *Society of Spanish Researchers in the United Kingdom/Comunidad de Científicos Españoles en el Reino Unido* (SRUK/CERU), *Científicos Españoles en la República Federal de Alemania*. (CERFA), *Asociación de Científicos Españoles en Japón* (ACE Japón), *Españoles Científicos en Estados Unidos* (ECUSA), *Asociación de Científicos Españoles en Suecia/Association of Spanish Scientists in Sweden* (ACES/ FSFS), *Spanish Researchers in Australia-Pacific/Investigadores Españoles en Australia-Pacífico* (SRAP/IEAP) and *Spanske Forskere i Danmark/Científicos Españoles en Dinamarca* (CED). The total SSA population from these associations at the time of this study was 1520, and their response rate was 16.51% (251). Additionally, *Fundación Universidad-Empresa* (FUE) has provided a young researchers list with 1158 scientists. Some other organizations helped to increase the number of addressees for this study: *Federación de Jóvenes Investigadores* (FJI), *Colegio Oficial de Físicos* (COFIS), *Federación Española de Biotecnólogos* (FEBiotec), *ARATECH* and *Centro de Innovación de la Universidad de Oviedo*. Overall, we have reached a YRS population of 2138 scientists with a response rate of 22.31% (477). For the SRS group, *Científicos Retornados a España* (CRE) and *Fundación Universidad-Empresa* (FUE) have provided us a total population of 302 scientists with a response rate of 27.48% (83). Our rates of response are similar to those by Barrufaldi and Landoni [20].

From December 18th, 2014 to April 30th, 2015 these associations and organizations have regularly distributed our survey among their researchers by e-mail. Approximately every two weeks, the authors have reported back the number of obtained responses to associations and entities. In mid-April, the last call for survey was sent and we have received very few answers since then. This survey was closed on April 30th, 2015.

### Questionnaire and data analysis

Our questionnaire followed Franzoni, Scellato and Stephan’s approach [19] (S1 Questionnaire–S3 Questionnaire) who have analyzed the mobility deciding factors of leading researchers from the 16 countries with the highest scientific production (excluding China): Australia, Belgium, Brazil, Canada, Denmark, France, Germany, India, Italy, Japan, Netherlands, Spain, Sweden, Switzerland, United Kingdom and United States. Provided with this knowledge base, ten variables were studied for the three selected groups in turn. These variables are grouped into three blocks as follows:

- Block 1 (*cumulative advantages*). This block is composed of five motivational scientific mobility variables in order to establish the differences between researchers, as reported by Merton [21, 22], with a special significance for the variable on the research career [23, 24]: (1) *opportunities to improve my career in the future* (*research career*), (2) *to improve my training* (*training*), (3) *greater availability of research funds* (*funding*), (4) *outstanding faculty*, *colleagues or research team* (*working with a world-class team*), and (5) *the prestige of the institution in the research area* (*institutional prestige*).
- Block 2 (*economic investments*). Two variables make up this block which focus on the economic investments to attract researchers into wealthy countries [9–11]: (6) *better wage and monetary compensation* (*wages*) and (7) *better facilities and infrastructure* (*facilities/infrastructure*).
- Block 3 (*social capabilities*). This block is composed of a set of variables related to the organizational effort in social aspects accessible to less-wealthy countries for attracting scientists. These variables allow us to compare (8) better *working conditions* (e.g., holidays, working hours), (9) better *fringe benefits* (e.g., parental leave, insurance, retirement pensions) *in the organization of the destination country* against (10) the inadequate level of *social responsibility in the organization of the departure country*. This latter variable includes different aspects such as low personal benefits, basic social and labor rights breaches, labor instability; and the lack of balance between work life and family life, environmental protection or measures to prevent of occupational hazards, to name a few.

For the SSA group, these variables refer to their decision to go abroad. For the SRS group, the variables refer to their decision to return. Since the YRS group has not left Spain, these variables are formulated as the possibility to leave in order to find Merton’s cumulative advantages in the future [21–24].

A Likert scale of 1 to 9 has been used to valuate these variables. Higher values mean a greater degree of importance; and lower values, a lesser degree. In order to interpret our results, the existing percentages between "important" (6) and "extremely important" (9) ratings have been considered.

Franzoni, Scellato and Stephan’s study [19] does not cover the participants’ profile as it only formulates a question about the scientists return possibility, with several answering options: “yes”, “no”, “depends on the job opportunities” or “perhaps part-time or at the end of career”. In our study, we firstly reformulate this question for SSA, SRS (in this case, as the possibility of leaving again), and YRS groups (as the possibility of going abroad). Secondly, we have followed Baruffaldi and Landoni’s approach [20] to expand their profile, including variables such as: the area of knowledge, gender, position in their organization and geographical location. According to all these variables, Baruffaldi and Landoni [20] concluded that their probability of return increases with their professional situation temporality and with other motives unrelated to employment opportunities. We also provide further data to deepen our understanding across different fields of knowledge.

### Survey development and properties

To ensure the survey quality, our procedure has followed these steps: (1) selection of the channel to get the participants’ responses, (2) selection and definition of the variables for the study, (3) instructions for participants, and (4) development of a pilot test for the draft survey.

This pilot test was delivered to a group of ten researchers from different fields. They were informed about the objectives and the study variables. Their answers allowed us to discuss the following aspects: (1) clarity of questions and variables, (2) the need to enhance the results by adding some more variables and (3) the need to eliminate redundant variables or any mismatch between them (leaving/ possibility of leaving for SSA and YRS and return for SRS).

As a result of this pilot, we have modified Franzoni, Scellato and Stephan’s [19] questionnaire. A new variable, named *to improve my training*, was added to represent the cumulative advantage as highlighted by Merton [20, 21]. Another variable related to social responsibility has been also included to assess the origin organization. This variable would additionally allow promoting scientific mobility into countries with scarce resources. On the other hand, those variables on which national actors have no influence have been removed such as: personal or family reasons, better quality of life, destination country lifestyle appeal, or international experience. Similarly, we have also removed the variable *lack of job opportunities in the country of residence* to prevent redundancy. Finally, the following series of variables with no equivalent in other scientific mobility types (going abroad / possibility to go abroad or return) were removed: *opportunity to extend my network of international relationships*, *visa or immigration reasons* and *opportunity to work with a specific academic group or colleagues*.

## Results

Our science mobility study is based on 811 responses to a survey of three different research groups: 477 from Young researchers in Spain (YRS), 251 from Spanish scientists abroad (SSA), and 83 from Spanish scientists returned to Spain (SRS).

First, we sought to identify the level of importance for scientific mobility of ten different variables divided in three blocks: cumulative advantages (variables 1–5), economic investments (variables 6–7), and social capabilities (variables 8–10). Table 1 shows the percentage of researchers indicating these variables as “important” or “extremely important”.

**Table 1:** Analysis of the importance of each of the ten variables per knowledge area and groups of research.

**Variable 1. Opportunities to improve my career in the future**			
	**Groups of Research (%)**^**a**^		
**Knowledge Area**	**YRS**^**b**^	**SSA**^**c**^	**SRS**^**d**^
Health Sciences	93.88	97.37	21.88
Social Sciences, Law, Arts and Humanities	94.22	90.00	42.86
Sciences	89.24	93.26	33.33
Engineering and Architecture	87.95	91.31	25.00
**Total**	**90.98**	**94.82**	**28.90**
**Variable 2. To improve my training**			
	**Groups of Research (%)**^**a**^		
**Knowledge Area**	**YRS**^**b**^	**SSA**^**c**^	**SRS**^**d**^
Health Sciences	79.59	92.99	15.62
Social Sciences, Law, Arts and Humanities	74.99	80.00	14.29
Sciences	78.98	88.45	22.22
Engineering and Architecture	71.08	73.92	37.50
**Total**	**77.35**	**88.84**	**20.47**
**Variable 3. Greater availability of research funds**			
	**Groups of Research (%)**^**a**^		
**Knowledge Area**	**YRS**^**b**^	**SSA**^**c**^	**SRS**^**d**^
Health Sciences	90.48	91.23	9.37
Social Sciences, Law, Arts and Humanities	75.00	80.00	14.29
Sciences	88.20	82.69	8.34
Engineering and Architecture	85.54	65.23	0.00
**Total**	**87.00**	**84.86**	**8.42**
**Variable 4. Outstanding faculty, colleagues or research team**			
	**Groups of Research (%)**^**a**^		
**Knowledge Area**	**YRS**^**b**^	**SSA**^**c**^	**SRS**^**d**^
Health Sciences	64.63	54.39	34.37
Social Sciences, Law, Arts and Humanities	71.15	80.00	28.58
Sciences	54.87	58.66	41.67
Engineering and Architecture	59.04	39.14	25.00
**Total**	**60.39**	**55.77**	**36.14**
**Variable 5. Excellence/prestige of the institution in my research area**			
	**Groups of Research (%)**^**a**^		
**Knowledge Area**	**YRS**^**b**^	**SSA**^**c**^	**SRS**^**d**^
Health Sciences	81.63	86.85	21.88
Social Sciences, Law, Arts and Humanities	78.84	90.00	28.58
Sciences	76.41	75.01	22.23
Engineering and Architecture	77.11	69.56	25.00
**Total**	**78.41**	**80.48**	**22.88**
**Variable 6. Better wage and monetary compensation**			
	**Groups of Research (%)**^**a**^		
**Knowledge Area**	**YRS**^**b**^	**SSA**^**c**^	**SRS**^**d**^
Health Sciences	91.16	76.31	12.50
Social Sciences, Law, Arts and Humanities	73.08	90.00	14.29
Sciences	85.64	75.00	8.34
Engineering and Architecture	89.16	86.95	0.00
**Total**	**86.58**	**77.30**	**9.62**
**Variable 7. Better facilities and infrastructures**			
	**Groups of Research (%)**^**a**^		
**Knowledge Area**	**YRS**^**b**^	**SSA**^**c**^	**SRS**^**d**^
Health Sciences	70.74	69.29	15.62
Social Sciences, Law, Arts and Humanities	57.69	70.00	14.29
Sciences	69.74	65.39	8.34
Engineering and Architecture	71.08	43.48	0.00
**Total**	**68.97**	**65.34**	**10.84**
**Variable 8. Better working conditions in the organization of the country of destination**			
	**Groups of Research (%)**^**a**^		
**Knowledge Area**	**YRS**^**b**^	**SSA**^**c**^	**SRS**^**d**^
Health Sciences	70.67	42.11	25.00
Social Sciences, Law, Arts and Humanities	59.61	30.00	14.29
Sciences	66.15	45.20	22.22
Engineering and Architecture	71.08	43.49	0.00
**Total**	**67.72**	**43.03**	**20.47**
**Variable 9. Better fringe benefits in the organization of the destination country**			
	**Groups of Research (%)**^**a**^		
**Knowledge Area**	**YRS**^**b**^	**SSA**^**c**^	**SRS**^**d**^
Health Sciences	46.94	21.05	18.75
Social Sciences, Law, Arts and Humanities	51.92	20.00	28.57
Sciences	53.34	24.04	30.55
Engineering and Architecture	54.22	17.40	0.00
**Total**	**51.36**	**21.91**	**22.88**
**Variable 10. Inadequate level of social responsibility in the organization of the departure country**			
	**Groups of Research (%)**^**a**^		
**Knowledge Area**	**YRS**^**b**^	**SSA**^**c**^	**SRS**^**d**^
Health Sciences	74.14	48.24	15.62
Social Sciences, Law, Arts and Humanities	63.46	60.00	42.86
Sciences	73.84	50.00	19.46
Engineering and Architecture	71.08	34.78	12.50
**Total**	**72.33**	**48.21**	**19.27**

^a^Percentage of researchers indicating "important" or "extremely important".

^b^YRS, Young researchers in Spain.

^c^SSA, Spanish scientists abroad.

^d^SRS, Spanish scientists returned to Spain.

### The importance of cumulative advantages for scientific mobility

The first variable studied focused on the *Research career* (Table 1, variable 1), most scientists in the Health Sciences YRS group (138 out of 147, 93.88%) consider the *opportunities to improve my career in the future* variable to be a decisive factor. This fact is also shown in the Health Sciences SSA group (111 over 114, 97.37%) for whom this variable has been crucial to their career progression, whereas only 21.88% (7 over 32) of the Health Sciences SRS group finds this variable crucial in returning from abroad. Ranked by knowledge areas, these percentages occupy the second, first and fourth positions, respectively. Regarding other areas of knowledge, it is important to note the relatively higher percentage of the SRS of Social Sciences, Law, Arts and Humanities for this variable.

In regards to the *Training* (Table 1, variable 2), a significant percentage of the Health Sciences YRS, close to 80% (117/147), considered that *to improve my training* is a crucial variable for going abroad (Table 1). It has also been a crucial variable for the 92.99% (106/114) of the Health Sciences SSA for leaving, whereas only 15.62% (5/32) of the Health Sciences SRS considered this variable as crucial for their return to Spain. As for all the knowledge areas, these percentages are rated as the first ones in the first two groups and rated as the third one in the Health Sciences SRS. It is interesting to note the high percentage of SRS in Engineering and Architecture (3/8, 37.50%), as reflected in this variable.

As for *Funding* (Table 1, variable 3), 90.48% (133/147) of the Health Sciences YRS considered the *greater availability of research funds* crucial for their departure abroad. The Health Sciences SSA share the same opinion (104/114, 91.23%). However, these percentages greatly decreased to 9.37% (3/32) in the case of the Health Sciences SRS. The latter percentage takes the second place in the classification by knowledge areas in this variable, while making the first place for the YRS and the SSA returns. It is worth noting that none of the SRS in Engineering and Architecture chose this variable as crucial for their return.

Regarding *Working with a world-class team* (Table 1, variable 4), 64.63% (95/147) of the Health Sciences YRS found it crucial to work with *outstanding faculty*, *colleagues or research team*. This relatively high percentage is reduced to 54.39% (62/117) in the case of the Health Sciences SSA. For the SRS in this area, the proportion is further reduced below 35% (11/32). The first and the third groups had the second position in relation to other knowledge areas, while the second group is in third position.

As for *Institutional prestige* (Table 1, variable 5), the *prestige of the institution* was important for the majority of Health Sciences YRS and SSA (120/147, 81.63% and 99/117, 86.85%, respectively). Similarly, these percentages dropped in the case of Health Sciences SRS (7/32, 21.87%). When considering knowledge areas, these percentages were in the first position for the case of YRS, in the second position for SSA and in the fourth position for SRS.

### The importance of economic investments for scientific mobility

When asked about *Wages* (Table 1, variable 6), 91.16% (134/147) of Health Sciences YRS considered that *better wage and monetary compensation* would be a crucial variable for going abroad. Similarly, it was an important reason for leaving the country for most of the Health Sciences SSA (87/114, 76.31%). For the Health Sciences SRS, this percentage decreased to 12.50% (4/32). In relation to the positions by knowledge areas, these percentages rated as first place for the YRS, third place for the SSA and second place for the SRS. Again, no SRS in Engineering and Architecture have considered this variable crucial for their return.

Regarding *Facilities/infrastructure* (Table 1, variable 7), more than 70% (104/147) of the Health Sciences YRS considered *better facilities and infrastructure* as a crucial variable for going abroad. This percentage is confirmed at similar levels (79/114, 69.29%) for the Health Sciences SSA group. Again, the percentage of Health Sciences SRS that considered this an important variable was reduced to 15.62% (5/32). As for the knowledge areas ratings, these percentages for Health Sciences occupy the second place in the first two groups and the first place for the SRS group. It should be noted that no SRS in Engineering and Architecture considered this variable crucial to returning.

### The importance of social capabilities for scientific mobility

In regards to the *Working conditions in the organization of the country of destination* (Table 1, variable 8), a percentage above 70% (104/147) of Health Sciences YRS considered *better working conditions* as a crucial variable for going abroad. This percentage drops to 42.11% (48/117) in the case of Health Sciences SSA. For the Health Sciences SRS, the percentage of those who believed that this variable was crucial for their return to Spain further drops to 25% (8/32). By knowledge area, the latter group rates in first position in percentage, whereas the YRS are in second place and the SSA, in third. As for some previous variables, no SRS in Engineering and Architecture decided to return to Spain because of this variable.

Regarding the *Fringe benefits in the organization of the destination country* (Table 1, variable 9), only 46.94% (69/147) of the Health Sciences YRS considered that the *better fringe benefits* to be gained abroad is a crucial variable for leaving Spain. This low percentage is similar for the SSA group in this area (24/117, 21.05%). The percentage for the Health Sciences SRS who considered these benefits crucial for returning to Spain is even lower (6/32, 18.75%). In relation to the positions in order of knowledge area for these groups in the Health Sciences, the YRS group occupies fourth place, the SSA second place, and the SRS third place. Again, none of the SRS in Engineering and Architecture decided to return to Spain according to this variable on personal performance.

Lastly, when the *Social responsibility in the organization of the departure country* was analyzed (Table 1, variable 10), for 74.14% (109/147) of the Health Sciences YRS, the *inadequate level of social responsibility offered by the organization in Spain* is a crucial variable for going abroad. This percentage is reduced to below 50% (55/117) for the Health Sciences SSA who considered this variable important for moving abroad. Only 15.62% (5/32) of the Health Sciences SRS answered positively to this variable as important for retuning to Spain. By knowledge areas, these percentages ranked in the third place for SSA and SRS, and in the first place for YRS.

### Participants’ profile

**Fig 1:**
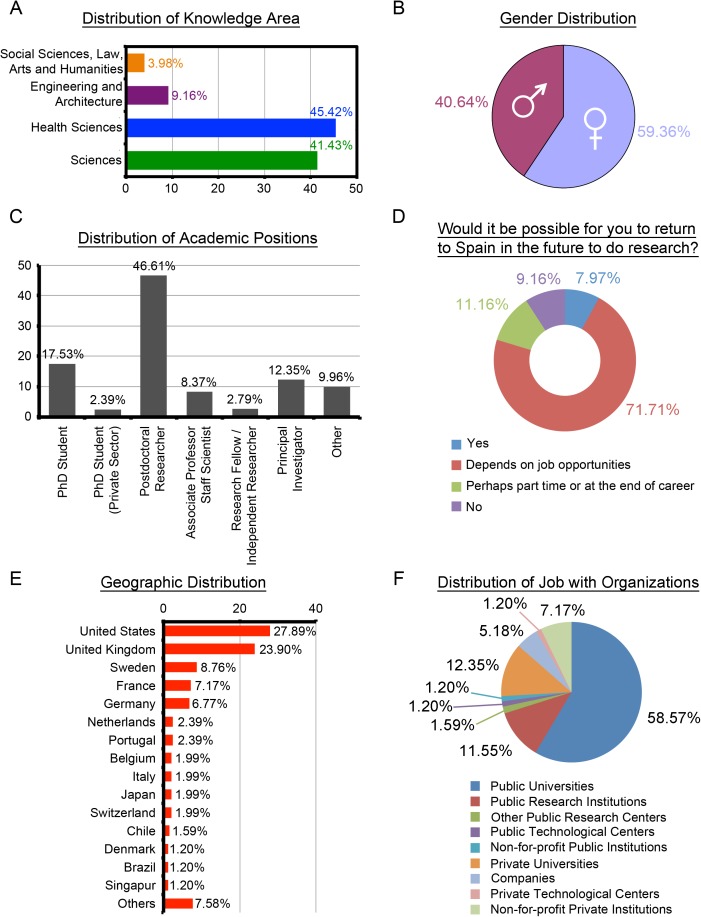
Profile of Spanish Scientists Abroad (n = 251). (A) Distribution of knowledge area in percentage. (B) Gender distribution in percentage. (C) Distribution of academic positions in percentage; by default, these positions are considered to be in the Public Sector unless otherwise stated. (D) Distribution of the opinion about a hypothetical return to Spain in percentages. (E) Distribution of geographic locations in the top-15 countries. Bottom-right is a map of World indicating countries; Others refers to "China (0.80%), Czech Republic (0.80%), Ireland (0.80%), Australia (0.40%), Bolivia (0.40%), Canada (0.40%), Costa Rica (0.40%), Ecuador (0.40%), Finland (0.40%), Mexico (0.40%), Reunion (0.40%), Slovenia (0.40%), South Africa (0.40%), South Korea (0.40%), United Arab Emirates (0.40%), No Response (0.40%)". (F) Distribution of job with organizations within the Public and Private Sectors.

The Health Sciences and Sciences knowledge areas represent more than 86% (218/251) of researchers in the SSA group (Fig 1A). As for gender participation, women account for 59.36% (149/251, Fig 1B). Most of the SSA scientists hold temporary positions in their organizations. Only 20.72% (52/251) have permanent positions either as Associate Professor or Scientific staff (8.37%), or as Principal Investigators (12.35%) at the Public Sector (Fig 1C). Also, an important 71.71% (180/251, Fig 1D) of the SSA would return to Spain depending on job opportunities. United States (70/251, 27.89%) and the UK (60/251, 23.90%,) are the chosen countries for most of the SSA group, followed at some distance by Sweden (22/251, 8.76%), which heads the group of other countries (Fig 1E). Both Public and Private Universities are typical institutions for more than 70% of the SSA (178/251), with Public Universities being preferred with a percentage higher than 58% (147/251, Fig 1F).

**Fig 2:**
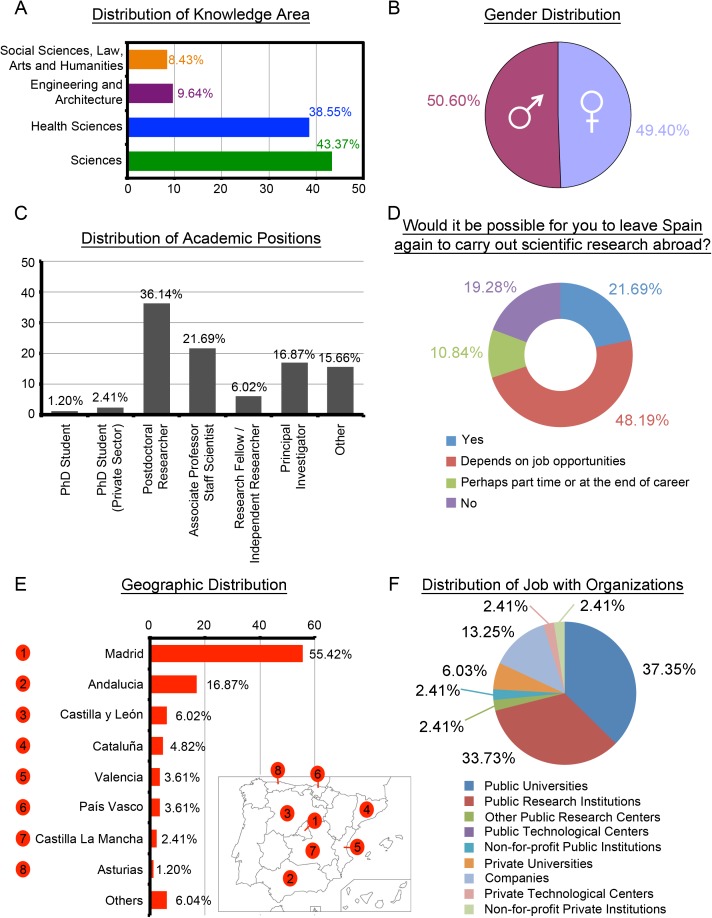
Profile of Scientists Returned to Spain (n = 83). (A) Distribution of knowledge areas in percentage. (B) Gender distribution in percentage. (C) Distribution of academic positions in percentage; by default, these positions are in the Public Sector unless otherwise stated. (D) Distribution of opinion about a hypothetical leaving Spain again in percentage. (E) Distribution of geographic locations in the top-8 autonomous regions. Bottom-right is a map of Spain indicating autonomous regions; Others refers to "Aragón (1.20%), Canarias (1.20%), Galicia (1.20%), Murcia (1.20%), Navarra (1.20%)". (F) Distribution of job with organizations within the Public and Private Sectors.

A percentage close to 82% (68/83, Fig 2A) of the SRS group belongs to the Sciences and Health Sciences knowledge areas. By gender, men represent 50.60% (42/83, Fig 2B). Only 38.56% of SRS (32/83) have a permanent position in their organization either as Associate Professors or Scientific Staff (21.69%, 18/83), or as Principal Investigators (16,87%, 14/83) in the Public Sector (Fig 2C). Besides, a significant percentage of 48.19% (40/83) of this group would leave depending on job opportunities (Fig 2D). According to Fig 2E, more than half of SRS work in the Community of Madrid (46/83, 55.42%), the rest are spread across the following communities: Andalucia (14/83, 16.87%), Castilla y León (5/83, 6.02%), Cataluña (4/83, 4.82%) and Valencia (3/83, 3.61%). This group’s most preferred organizations are Public Universities and Public Research Institutions, with more than 71% (59/83, Fig 2F).

**Fig 3:**
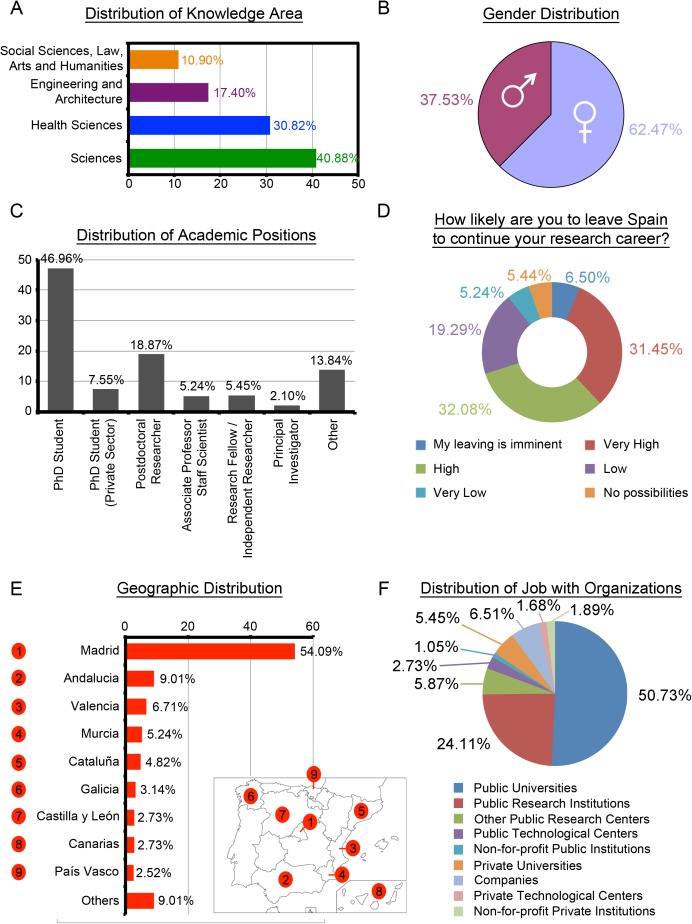
Profile of Young Researchers in Spain (n = 477). (A) Distribution of knowledge areas in percentage. (B) Gender distribution in percentage. (C) Distribution of academic positions in percentage; by default, these positions are considered to be in the Public Sector unless otherwise stated. (D) Distribution of the opinion about a hypothetical departure from Spain in percentages. (E) Distribution of geographic locations in the top-9 indicating autonomous regions. Bottom-right is a map of Spain with autonomous regions; Others refers to "Aragón (1.26%), Asturias (2.31%), Cantabria (0.63%), Castilla La Mancha (2.10%), Extremadura (1.05%), Navarra (0.63%), La Rioja (0.42%), No Response (0.63%)". (F) Distribution of jobs with organizations within the Public and Private Sectors.

Likewise, most of the YRS participants in the study belong to the Sciences and Health Sciences knowledge areas (342/477, 70%, Fig 3A). Regarding gender, there has been a major response from women with 62.47% (298/477, Fig 3B). Only 7.34% (35/477) of the YRS have a permanent position either as Associate Professors or Scientific Staff (5.24%), or Principal Investigators (2.10%) at the Public Sector (Fig 3C). Similarly for over 70% of this group (334/477), the likelihood of going abroad in order to continue their research career is extremely high (Fig 3D). As in the case of SRS, more than half of YRS (258/477, 54.09%) belonged to the Community of Madrid, followed far behind by other communities with much lower participation rates: Andalucia, Valencia, Murcia and Cataluña (Fig 3E). Also, most researchers in this group belong to Public Universities and Public Research Institutions with a significant percentage of 74.84% (357/477, Fig 3F).

**Fig 4:**
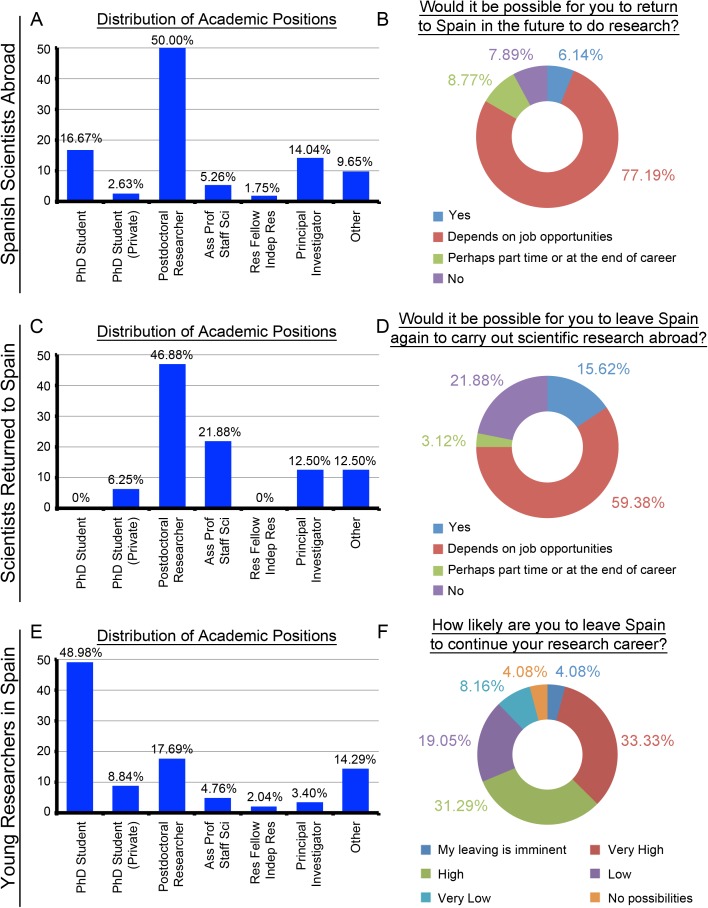
Distribution of Academic Positions and Possibilities of scientific circulation: Health Sciences (n/SSA = 114; n/SRS = 32; n/YRS = 147). (A) Distribution of Academic Positions for SSA. (B) Would it be possible for you to return to Spain in the future to do research? SSA group. (C) Distribution of Academic Positions for SRS. (D) Would it be possible for you to leave Spain again to carry out scientific research abroad? SRS group. (E) Distribution of Academic Positions for YRS. (F) How likely are you to leave Spain to continue your research career? YRS group.

**Fig 5:**
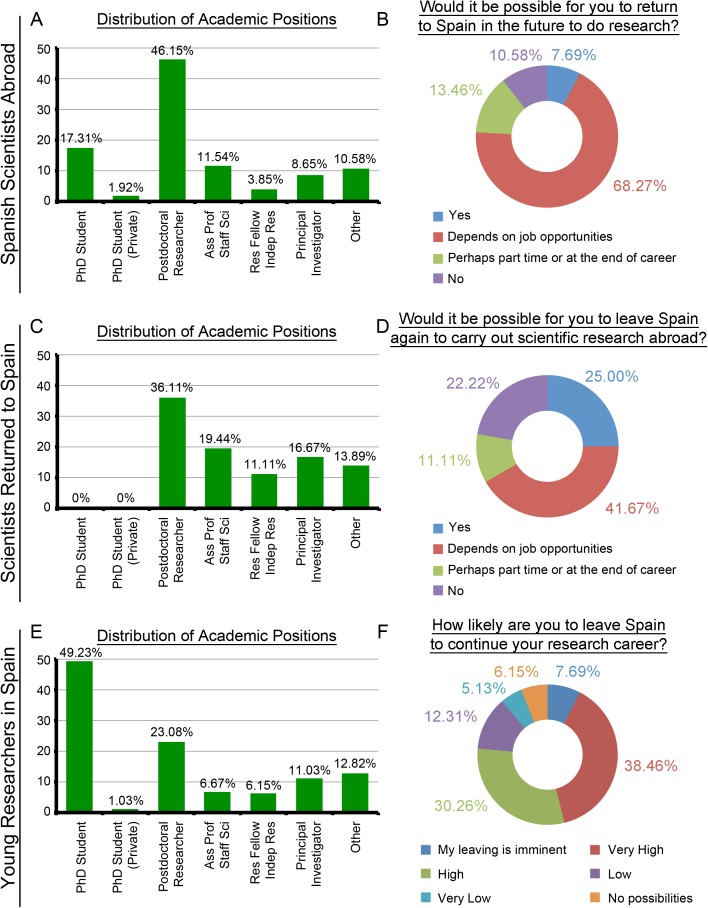
Distribution of Academic Positions and Possibilities of scientific circulation: sciences (n/SSA = 104; n/SRS = 36; n/YRS = 195). (A) Distribution of Academic Positions for SSA. (B) Would it be possible for you to return to Spain to do research in the future? SSA group. (C) Distribution of Academic Positions for SRS. (D) Would it be possible for you to leave Spain again to carry out scientific research abroad? SRS group. (E) Distribution of Academic Positions for YRS. (F) How likely are you to leave Spain to continue your research career? YRS group.

There are some remarkable facts in connection with these aspects for all knowledge areas. In the case of starting a new mobility action, the Health Sciences SSA who would come back to Spain according to job opportunities, the Health Sciences SRS who would move abroad again depending on job opportunities and the Sciences YRS with a high likelihood of leaving Spain in order to foster their research career show the highest percentages (with 77.19%, 88/114, Fig 4B; 59.38%, 19/32, Fig 4D; and 76.41%, 149/195, Fig 5F, respectively). With regard to their permanent position, the number of researchers is generally small. It is only necessary to highlight the important percentages of the SSA in Social Sciences, Law, Arts and Humanities (6/10, 60%, S1A Fig), SRS in Social Sciences, Law, Arts and Humanities (4/7, 57.14%, S1C Fig) and SRS in Engineering and Architecture. (4/8, 50%, S2C Fig).

## Discussion

Our results show that for the Health Sciences YRS and SSA the most crucial variables for returning to Spain are basically those related to the cumulative advantages explained by Merton [21, 22]. In the case of the Health Sciences SRS, in addition to these cumulative advantages, there also are other variables related to social capabilities that mainly explain their return to Spain.

For most of the Health Sciences YRS, their main variables to explain leaving are predominantly related to the *cumulative advantages* (except *working with in a world-class team*) plus *wages* (see 5 top positions in S1 Table). This concentration on these *cumulative advantages* occurs more clearly for a large majority in Health Sciences SSA as crucial variables for leaving, because wages in this case rank fifth (S1 Table). This is in agreement with the previous study by Franzoni, Scellato and Stephan [19], as researchers’ reasons to leave were also focused on *cumulative advantages*, especially in the case of *research career* (first position), *institutional prestige* (third position) and *funding* (fifth position). Additionally, other important variables were other *cumulative advantages* such as *working with a world-class team* (second position) or *facilities/infrastructure* (fourth position). This may be due to the selection of countries in this study [19], as it included countries with many resources [9–11] such as the US, Japan, UK, Germany, France, Sweden and Denmark, where researchers would likely be able to find Merton’s *cumulative advantages* [21, 22] without leaving. In the Spanish case of health sciences, these cumulative advantages are less covered, as discussed above in *research career* [34–38, 40–43], *funding* [39, 41–43], *training* [41–43], *institutional prestige* [43] and *working with a world-class team*. In any case, in the two studies it is confirmed that the research career is the primary variable for going abroad. This importance of the research career is consistent with previously mentioned studies [23, 24, 44].

By areas of knowledge, Health Sciences YRS is the main group that considers the variable training, funding, wages, institutional prestige and social responsibility as crucial for their potential departure (S2 Table). Also, Health Science SSA, is the main group that considers research career, training and funding to be among these crucial variables (S2 Table). These data reveal a specific need for improvement with respect to other knowledge areas and other crucial variables such as wages and social responsibility offered in Spain.

As opposed to Health Sciences YRS and SSA groups, it is difficult for the SRS scientists in this knowledge area to find critical variables for explaining their decision (S1 Table). Within the variables in the first positions, there are three of Merton’s cumulative advantages [21, 22] such as *working with a world-class team*, research career and institutional prestige. However, other cumulative advantages such as training and funding are at the bottom of the ranking (S1 Table). The highest positions are covered by variables related to social capabilities such as working conditions and fringe benefits in the destination country (S1 Table). This fact suggests that Spain may somehow balance its difficulties in delivering cumulative advantages by providing social capabilities to the Health Sciences SRS.

Franzoni, Scellato and Stephan’s study [19] agrees on mostly the same variables (save for the facilities/infrastructure) to explain the Health Sciences SRS case, with a percentage of researchers also below 50%. In their study, it is worth noting that research career takes the first place. When Franzoni, Scellato and Stephan refer to Spanish researchers, their study shows percentages similar to those in ours and indicates the same decision variables; they also rate the research career in the first place. The reason may be that career conditions in Spain have deteriorated since the year of their publication. Note that this fact is also consistent with other considerations by the most recent studies cited above [37–38].

Framing the Health Sciences among the different knowledge areas may allow us to assess its situation. Health Sciences SRS consider the research career and the institutional prestige in the lowest position within the key variables (S2 Table). This opinion reveals the need to improve on these aspects which are also considered as key elements by YRS and SSA groups. None of the scientists in the Engineering and Architecture SRS decided to return because of the variables facilities/infrastructure, funding, wages, working conditions in the country of destination and fringe benefits of the destination country (see S2 Table). Percentages much higher to the rest of the knowledge areas in the SRS of Social Sciences, Law, Arts and Humanities for variable research career and for the SRS in Engineering and Architecture for variable training, have also been observed (S2 Table). These results suggest that it might be interesting to pursue these sorts of measurements for these variables across different areas in order to come up with some good practices applicable to the Health Sciences.

As previously mentioned regarding the work of Baruffaldi and Landoni [20], the probability of return increases with a temporary professional status and mobility reasons unrelated to improving employment opportunities. According to this, in the case of the SSA in Health Sciences the percentage of researchers with a permanent position is quite low and the proportion of researchers who could return to Spain depending on job opportunities is very high. The percentage of the YRS in Health Sciences with a permanent position is even lower and many of them have high potential to go abroad to continue their research career.

Our data suggest that there is space for the Spanish administrations and healthcare organizations to formulate attractive policies for these groups. Although an significant batch of European funds (92.5 million euros from the ERC and 61.9 million euros from the MSCA) [52] have already been transferred to policies to attract researchers, there is still some potential for improvement in this direction by the Spanish administrations. Additionally, the tracking and analysis of the policies already set, such as the *Miguel Servet Programme* by the *Sistema Nacional de Salud* (SNS) [3] or the *Acción Estratégica de Salud* (AES) by the *Spanish National Plan for R&D&i 2013–2016* [43], may allow us to detect possible improvements for the researchers’ careers. Likewise, although the cumulative advantages offered by other countries are higher, Spanish healthcare organizations can establish collaborative policies ensuring the appropriate conditions for their return. As indicated above, in this aspect of the collaboration is still necessary for the Spanish organizations to make a greater effort [45] and there are a lot of associations of Spanish scientists abroad willing to intensify this cooperation.

Taking into account Baruffaldi and Landoni’s work [20] and according to our results, there is also a margin to create retention policies for the Health Sciences SRS, as only a few of them have a permanent position and many would go abroad again depending on job opportunities. This work implies that the *social capabilities* are complementary variables for SRS to return. Public-private collaborations by Spanish healthcare organizations may foster these capabilities. This collaboration is able to provide the SRS with the adequate working conditions and fringe benefits (payments of social insurance or kinder gardens, for example). This way it may be possible to compete with wealthier countries’ health organizations in offering SRS the cumulative advantages. This collaboration not only allows the transfer of scientific knowledge to the institution, enhancing its prestige, but also improves researchers’ performance^5^ and their career by participating in translational research.

Other measures can supplement public-private collaborations. Among them, the employment of researchers in hospitals and companies (pharmaceutical companies, for example) or supporting these SRS to launch entrepreneurship ventures can be particularly interesting because, since just a few of SRS work in the business sector, these measures would support translational research. The fact that many Spanish SRS are somehow linked to Madrid, where many Health organizations (hospitals, laboratories, new biotechnology companies) are located, can help in this endeavor [53]. Additionally, since many women have returned to Spain, equality plans in companies may contribute to their establishment.

Evidence provided by this work may contribute to improving the National Public Health Services, as the adequate mobility of researchers can enable new scientific knowledge to be brought into clinical practice. In addition, vigorous research in Health Sciences would help support the authorities in a more effective and efficient management of Health services. New drugs may also be a beneficial outcome of this imported research capability as well as new vaccines, medical devices or equipment with the potential to improve the diagnosis and treatment of patients and become a new source of employment and wealth creation.

### Limitations and future research

Data obtained throughout this study are reliable and consistent with other research. However, since the number of Spanish researchers in Health Sciences reached is rather small, further studies extending this number are desirable. The lack of knowledge on the total population for the three groups of researchers studied has made necessary the use of data from different associations and institutions. Because of this limitation, our results are unlikely to apply to the whole population of scientists for these groups.

This study suggests effective policies to articulate scientific mobility in the Health area at a given time, but does not go into its evolution. Therefore, future work should extend the time horizon by a longitudinal design.

Finally, our results strongly point to public-private partnerships and support for entrepreneurship in the Health Area as the most promising subjects for future research.

### Conclusions

This study provides data that show that the cumulative advantages are the crucial variables to explain why Spanish scientists in Health Sciences leave Spain. However in order to influence their return, these variables can be complemented by the social capabilities. These results suggest that the Spanish Health organizations can make Spain more attractive for researchers by means of promoting their social capabilities besides job opportunities or other cumulative advantages offered by wealthier countries. Researchers may then fulfill their role for the benefit of the National Public Health and help to achieve a balance between local and global Science.

## Supporting information

S1 Questionnaire Questionnaire for Spanish researchers abroad-Cuestionario para los científicos españoles en el exterior. (DOC)

S2 Questionnaire Questionnaire for scientists returned to Spain-Cuestionario para los científicos retornados a España. (DOC)

S3 Questionnaire Questionnaire for young researchers in Spain-Cuestionario para los los jóvenes investigadores en el España. (DOC)

S1 File Spanish scientists abroad-Científicos españoles en el exterior. (ODS)

S2 File Scientists returned to Spain-Científicos retornados a España. (ODS)

S3 File Young researchers in Spain-Jóvenes investigadores en España. (ODS)

S4 File Spanish scientists abroad. Sciences-Científicos españoles en el exterior. Ciencias.
(ODS)

S5 File Scientists returned to Spain. Sciences-Científicos retornados a España. Ciencias.
(ODS)

S6 File Young researchers in Spain. Sciences-Jóvenes investigadores en España. Ciencias.
(ODS)

S7 File Spanish scientists abroad. Health Sciences-Científicos españoles en el exterior. Ciencias de la salud.
(ODS)

S8 File Scientists returned to Spain. Health Sciences-Científicos retornados a España. Ciencias de la salud.
(ODS)

S9 File Young researchers in Spain. Health Sciences-Jóvenes investigadores en España. Ciencias de la salud.
(ODS)

S10 File Spanish scientists abroad. Social Sciences, Law, Arts and Humanities-Científicos españoles en el exterior. Ciencias sociales, jurídicas, artes y humanidades.
(ODS)

S11 File Scientists returned to Spain. Social Sciences, Law, Arts and Humanities-Científicos retornados a España. Ciencias sociales, jurídicas, artes y humanidades.
(ODS)

S12 File Young researchers in Spain. Social Sciences, Law, Arts and Humanities-Jóvenes investigadores en España. Ciencias sociales, jurídicas, artes y humanidades.
(ODS)

S13 File Spanish scientists abroad. Engineering and Architecture-Científicos españoles en el exterior. Ingeniería y arquitectura.
(ODS)

S14 File Scientists returned to Spain. Engineering and Architecture-Científicos retornados a España. Ingeniería y arquitectura.
(ODS)

S15 File Young researchers in Spain. Engineering and Architecture-Jóvenes investigadores en España. Ingeniería y arquitectura.
(ODS)

S1 Fig Distribution of Academic Positions and Possibilities of scientific mobility: social sciences, law, arts and humanities (n/SSA = 10; n/SRS = 7; n/YRS = 52). (A) Distribution of Academic Positions for SSA. (B) Would it be possible for you to return to Spain in the future to do research? SSA group. (C) Distribution of Academic Positions for SRS. (D) Would it be possible for you to leave Spain again to carry out scientific research abroad? SRS group. (E) Distribution of Academic Positions for YRS. (F) How likely are you to leave Spain to continue your research career? YRS group.
(TIF)

S2 Fig Distribution of Academic Positions and Possibilities of scientific mobility: engineering and architecture (n/SSA = 23; n/SRS = 8; n/YRS = 83). (A) Distribution of Academic Positions for SSA. (B) Would it be possible for you to return to Spain in the future to do research? SSA group. (C) Distribution of Academic Positions for SRS. (D) Would it be possible for you to leave Spain again to carry out scientific research abroad? SRS group. (E) Distribution of Academic Positions for YRS. (F) How likely are you to leave Spain to continue your research career? YRS group.
(TIF)

S1 Table Data on crucial variables for researchers in Health Sciences. (DOC)

S2 Table Data on crucial variables for knowledge areas: maximum percentage-minimum percentage. (DOC)

## Abbrevations

ACE Japón: Asociación de Científicos Españoles en Japón
ACES: Association of Spanish Scientists in Sweden/Asociación de Científicos Españoles en Suecia
AES: Acción Estratégica de Salud
CED: Spanske Forskere i Danmark/Científicos Españoles en Dinamarca
CERFA: Científicos Españoles en la República Federal de Alemania
COFIS: Colegio Oficial de Físicos
ECUSA: Comunidad de Españoles Científicos en Estados Unidos
FEBiotec: Federación Española de Biotecnólogos
FECYT: Spanish Foundation for Science and Technology /Fundación Española para la Ciencia y la Tecnología
FUE: Fundación Universidad-Empresa
MEC: Ministerio de Educación y Ciencia
R&D&i: Research, Development and Innovation
RedIEX: Sistema de Comunicación con Investigadores en el Exterior
SECTI: Sistema Español de Ciencia y Tecnología
SNS: Sistema Nacional de Salud
SRAP/IEAP: Spanish Researchers in Australia-Pacific/Investigadores Españoles en Australia-Pacífico
SRS / CRE: Scientists returned to Spain / Científicos retornados a España
SRUK/CERU: Society of Spanish Researchers in the United Kingdom/Comunidad de Científicos Españoles en el Reino Unido
SSA / CIEX: Spanish scientists abroad / Científicos españoles en el exterior
YRS / JIES: Young researchers in Spain / Jóvenes investigadores en España
