# Supplementary material for: Cumulative advantages and social capabilities in scientific mobility in the Health Sciences: The Spanish case
Source: PLoS One. 2017 Mar 15;12(3):e0173204. doi: 10.1371/journal.pone.0173204 (PMC5351855; doi:10.1371/journal.pone.0173204)
Supplement: S3 Questionnaire — (DOC) [file pone.0173204.s003.doc]

**Cuestionario para los jóvenes investigadores en España**

Questionnaire for Young researchers in Spain

| **Indique el tipo de organización donde realiza su labor investigadora principal.**  Indicate the type of organization where you perform your main research work. | | | | | |
| --- | --- | --- | --- | --- | --- |
| **Opción**  Answer | **Cuenta**  Account | **Porcentaje**  Percentage | | | |
| **Universidad pública (1)**  Public University (1) |  |  | | | |
| **Universidad privada (2)**  Private University (2) |  |  | | | |
| **Organismo público de investigación (3)**  Public Research Institution (3) |  |  | | | |
| **Otros centros de I+D de las Administraciones Públicas (4)**  Other Public Research Centers (4) |  |  | | | |
| **Centro tecnológico de propiedad y gestión pública (5)**  Public Technological Center (5) |  |  | | | |
| **Centro tecnológico de propiedad y gestión privada (6)**  Private Technological Center (6) |  |  | | | |
| **Entidad pública sin ánimo de lucro (7)**  Non-for-profit Public Institution (7) |  |  | | | |
| **Entidad privada sin ánimo de lucro (8)**  Non-for-profit Private Institution (8) |  |  | | | |
| **Gran empresa (igual o superior a 250 empleados) (9)**  Large company (equal or more than 250 employees) (9) |  |  | | | |
| **Mediana empresa (menos de 250 empleados) (10)**  Medium company (less than 250 employees) (10) |  |  | | | |
| **Pequeña empresa (menos de 50 empleados) (11)**  Small company (less than 50 employees) (11) |  |  | | | |
| **Microempresa (menos de 10 empleados) (12)**  Microcompany (less than 10 employees) (12) |  |  | | | |
| **Señale el área de conocimiento objeto de esta labor investigadora.**  Select the area of ​​knowledge for your research work. | | |  |  |  |
| **Opción**  Answer | **Cuenta**  Account | **Porcentaje**  Percentage |  |  |  |
| **Ciencias (1)**  Sciences (1) |  |  |  |  |  |
| **Ciencias de la Salud (2)**  Health Sciences (2) |  |  |  |  |  |
| **Ciencias Sociales, Jurídicas, Artes y Humanidades (3)**  Social sciences, Law, Arts and Humanities (3) |  |  |  |  |  |
| **Ingeniería y Arquitectura (4)**  Engineering and Architecture (4) |  |  |  |  |  |

| **Seleccione Comunidad o Ciudad Autónoma en caso de haber elegido España en la pregunta anterior**  Have you chosen Spain in the previous question, please select Region or Autonomous City as the answer below | | |
| --- | --- | --- |
| **Opción**  Answer | **Cuenta**  Account | **Porcentaje**  Percentage |

| **Identifique el grupo al que pertenece dentro del colectivo investigador:**  **Identify your research collective** | | |
| --- | --- | --- |
| **Opción**  Answer | **Cuenta**  Account | **Porcentaje**  Percentage |
| **Investigador predoctoral del sector público (1)**  PhD Student at  the Public Sector (1) |  |  |
| **Investigador postdoctoral del sector público (2)**  Postdoctoral Researcher at the Public Sector (2) |  |  |
| **Investigador contratado doctor o científico de plantilla del sector público (3)**  Associate Professor or Scientific Staff at the Public Sector (3) |  |  |
| **Investigador predoctoral del sector privado (4)**  PhD Student at the Private Sector (4) |  |  |
| **Investigador independiente (5)**  Research Fellow/Independent Researcher (5) |  |  |
| **Investigador titular (lecturer, senior lecturer, reader, professor, team leader) (6)**   Principal Investigator (Lecturer, Senior Lecturer, Reader, Professor, Team Leader) (6) |  |  |
| **Otro (7)**  Other (7) |  |  |

| **Sexo:**  **Gender:** | | |
| --- | --- | --- |
| **Opción**  Answer | **Cuenta**  Account | **Porcentaje**  Percentage |
| **Femenino (F)**  Female (F) |  |  |
| **Masculino (M)**  Male (M) |  |  |

| **¿Cuáles son las posibilidades de que Usted se marche al exterior para continuar con su carrera investigadora?**  How likely are you to leave Spain to continue your research career? | | |
| --- | --- | --- |
| **Opción**  Answer | **Cuenta**  Account | **Porcentaje**  Percentage |
| **Mi marcha es inminente (1)**  My leaving is imminent (1) |  |  |
| **Muy altas (2)**  Very high (2) |  |  |
| **Altas (3)**  High (3) |  |  |
| **Escasas (4)**  Low (4) |  |  |
| **Muy escasas (5)**  Very Low (5) |  |  |
| **Nulas (6)**  No possibilities (6) |  |  |

| **¿En qué medida son importantes los siguientes factores en su decisión de marchar al exterior para continuar con su carrera investigadora?**  **Escala de Likert de 1 a 9: nada importante = 1; neutral = 5; extremadamente importante = 9.**  To what extent are the following factors important in your decision to go abroad in order to continue your research career? Likert scale from 1 to 9: Totally unimportant = 1; Neutral = 5; 9 = Extremely important. | | |
| --- | --- | --- |
| **Opción**  Answer | **Cuenta**  Account | **Porcentaje**  Percentage |
| **Oportunidades para mejorar mis perspectivas de carrera en el futuro**  Opportunities to improve my career in the future |  |  |
| **Excepcionales profesores, compañeros de trabajo o miembros del equipo de investigación**  Outstanding faculty, colleagues or research team |  |  |
| **Mejores instalaciones e infraestructuras**  Better facilities and infrastructures |  |  |
| **Abundantes fondos económicos para la investigación**  Greater availability of research funds |  |  |
| **Excelencia/prestigio de la institución extranjera en mi área de investigación**  Excellence/prestige of the institution in my area of research |  |  |
| **Mejor salario y compensaciones monetarias**  Better wage and monetary compensation |  |  |
| **Mejores condiciones laborales (vacaciones, horas de trabajo, por ejemplo)**  Better working conditions (holidays, working hours, for example) |  |  |
| **Mejores prestaciones personales (permisos parentales, pensiones, seguros, entre otros)**  Better fringe benefits (parental leave, insurance, retirement pensions, among other aspects) |  |  |
| **Mejorar mi formación**  To improve my training |  |  |
| **Nivel inadecuado de responsabilidad social en mi organización en España (inexistencia de medidas de conciliación profesional y familiar, inestabilidad laboral, bajas prestaciones personales, incumplimiento de derechos sociolaborales básicos, entre otros aspectos)**  Inadequate level of social responsibility in my organization in Spain (the lack of balance between work life and family life, labor instability, low personal benefits, basic social and labor rights breaches, among other aspects) |  |  |
