# Supplementary figures and images for: Cumulative advantages and social capabilities in scientific mobility in the Health Sciences: The Spanish case
Source: PLoS One. 2017 Mar 15;12(3):e0173204. doi: 10.1371/journal.pone.0173204 (PMC5351855; doi:10.1371/journal.pone.0173204)

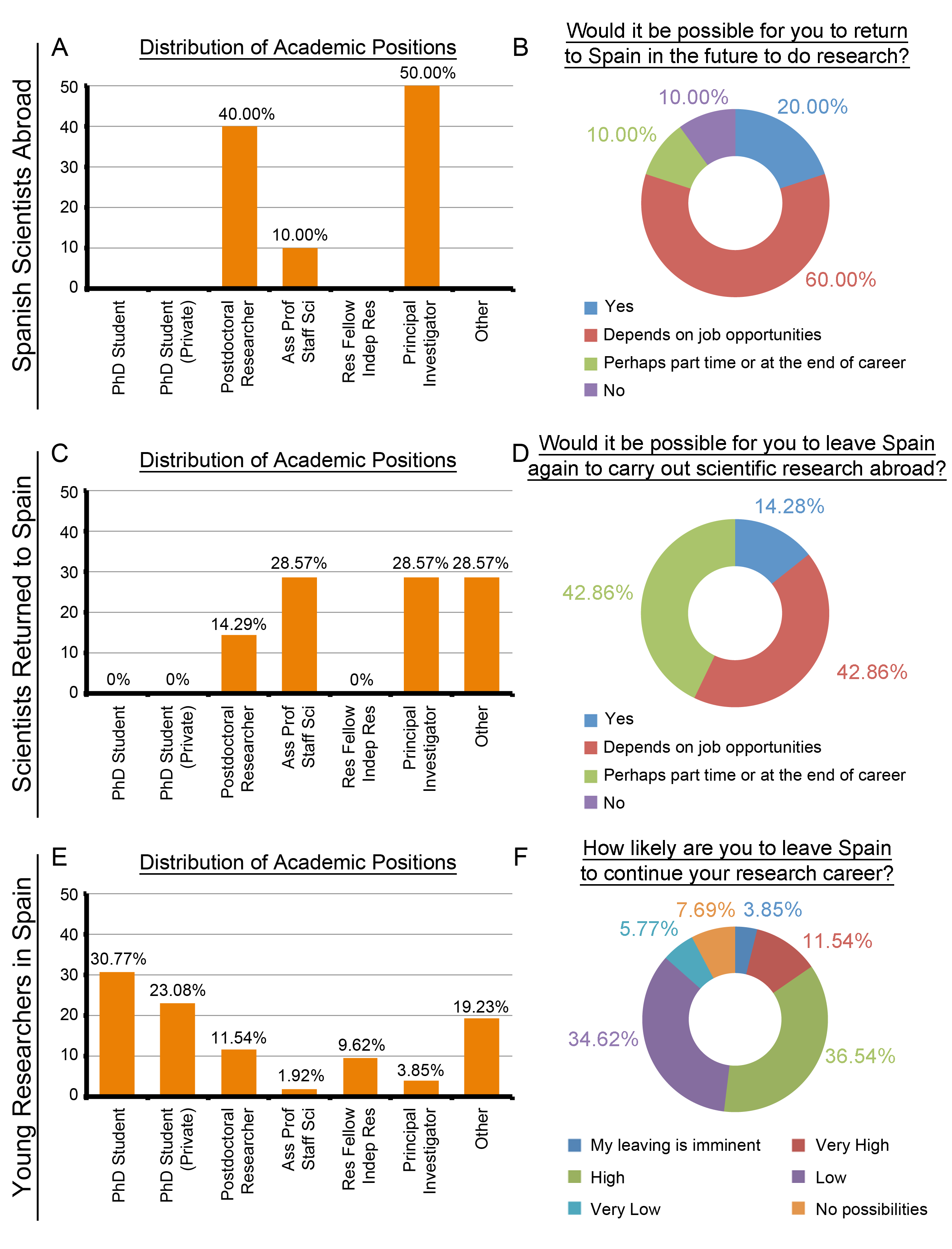

Supplement: S1 Fig — (A) Distribution of Academic Positions for SSA. (B) Would it be possible for you to return to Spain in the future to do research? SSA group. (C) Distribution of Academic Positions for SRS. (D) Would it be possible for you to leave Spain again to carry out scientific research abroad? SRS group. (E) Distribution of Academic Positions for YRS. (F) How likely are you to leave Spain to continue your research career? YRS group. (TIF) [file pone.0173204.s019.tif]

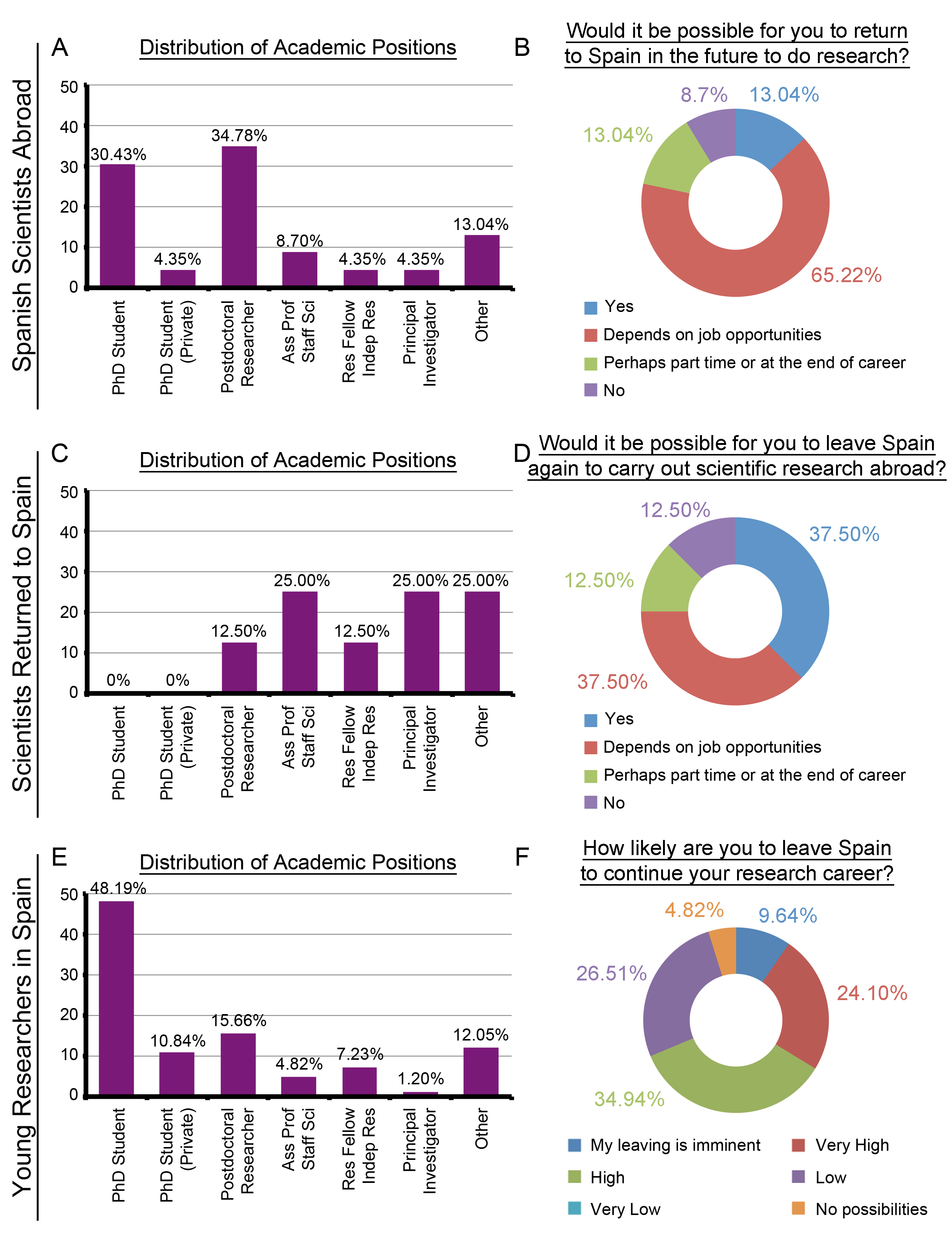

Supplement: S2 Fig — (A) Distribution of Academic Positions for SSA. (B) Would it be possible for you to return to Spain in the future to do research? SSA group. (C) Distribution of Academic Positions for SRS. (D) Would it be possible for you to leave Spain again to carry out scientific research abroad? SRS group. (E) Distribution of Academic Positions for YRS. (F) How likely are you to leave Spain to continue your research career? YRS group. (TIF) [file pone.0173204.s020.tif]
