# Supplementary material for: Cumulative advantages and social capabilities in scientific mobility in the Health Sciences: The Spanish case
Source: PLoS One. 2017 Mar 15;12(3):e0173204. doi: 10.1371/journal.pone.0173204 (PMC5351855; doi:10.1371/journal.pone.0173204)
Supplement: S1 Table — (DOC) [file pone.0173204.s021.doc]

**S1 Table. Data on crucial variables for researchers in Health Sciences**

| **Position** | **YRS** | | | **SSA** | | | **SRS** | |
| --- | --- | --- | --- | --- | --- | --- | --- | --- |
|  | **Variables** | **%** | **Variables** | | **%** | **Variables** | | **%** |
| **1^st^** | Research career | 93.88% | Research career | | 97.37% | Working with a world-class teams | | 34.37% |
| **2^nd^** | Wages | 91.64% | Training | | 92.99% | Working conditions in the organization of the destination country | | 25.00% |
| **3^rd^** | Funding | 90.48% | Funding | | 91.23% | Research career | | 21.88% |
| **4^th^** | Institutional Prestige | 81.63% | Institutional Prestige | | 86.85% | Institutional Prestige | | 21.88% |
| **5^th^** | Training | 79.59% | Wages | | 76.31% | Fringe benefits in the organization of the destination country | | 18.75% |
| **6 ^th^** | Social responsibility in the organization of the departure country | 74.14% | Facilities/  infrastructure | | 69.29% | Training | | 15.62% |
| **7 ^th^** | Facilities/  infrastructure | 70.74% | Working with a world-class teams | | 54.39% | Social responsibility in the organization of the departure country | | 15.62% |
| **8 ^th^** | Working conditions in the organization of the country of destination | 70.67% | Social responsibility in the organization of the departure country | | 48.24% | Facilities/  infrastructure | | 15.62% |
| **9 ^th^** | Working with a world-class teams | 64.63% | Working conditions in the organization of the destination country | | 42.11% | Wages | | 12.50% |
| **10^th^** | Fringe benefits in the organization of the destination country | 46.94% | Fringe benefits in the organization of the destination country | | 21.05% | Funding | | 9.37% |
