# Supplementary material for: Cumulative advantages and social capabilities in scientific mobility in the Health Sciences: The Spanish case
Source: PLoS One. 2017 Mar 15;12(3):e0173204. doi: 10.1371/journal.pone.0173204 (PMC5351855; doi:10.1371/journal.pone.0173204)
Supplement: S2 Table — (DOC) [file pone.0173204.s022.doc]

**S2 Table. Data on crucial variables for knowledge areas: maximum percentage / minimum percentage**

| **Crucial variable** | **YRS**  **(maximum percentage / minimum percentage)** | **SSA**  **maximum percentage / minimum percentage** | **SRS**  **maximum percentage / minimum percentage** |
| --- | --- | --- | --- |
| **Research career** | Social Sciences, Law, Arts and Humanities (94.22%) | Health Sciences (97.37%) | Social Sciences, Law, Arts and Humanities (42.86%) |
|  | Engineering and Architecture (87.95%) | Engineering and Architecture (91.31%) | Health Sciences (21.88%) |
| **Training** | Health Sciences (79.59%) | Health Sciences (92.99%) | Engineering and Architecture (37.50%) |
|  | Engineering and Architecture (71.08%) | Engineering and Architecture (73.92%) | Social Sciences, Law, Arts and Humanities (14.29%) |
| **Funding** | Health Sciences (90.48%) | Health Sciences (91.23%) | Social Sciences, Law, Arts and Humanities (14.29%) |
|  | Social Sciences, Law, Arts and Humanities (75.00%) | Engineering and Architecture (65.23%) | Engineering and Architecture (0.00%) |
| **Working with a world-class teams** | Social Sciences, Law, Arts and Humanities (71.15%) | Social Sciences, Law, Arts and Humanities (80.00%) | Sciences  (41.67%) |
|  | Sciences  (54.87%) | Engineering and Architecture (39.14%) | Engineering and Architecture (25.00%) |
| **Institutional prestige** | Health Sciences (81.63%) | Social Sciences, Law, Arts and Humanities (90.00%) | Social Sciences, Law, Arts and Humanities (28.58%) |
|  | Sciences (76.41%) | Sciences (75.01%) | Health Sciences (21.87%) |
| **Wages** | Health Sciences (91.16%) | Social Sciences, Law, Arts and Humanities (90.00%) | Social Sciences, Law, Arts and Humanities (14.29%) |
|  | Social Sciences, Law, Arts and Humanities (73.08%) | Sciences  (75.00%) | Engineering and Architecture (0.00%) |
| **Facilities/infrastructure** | Engineering and Architecture (71.08%) | Social Sciences, Law, Arts and Humanities (70.00%) | Health Sciences (15.62%) |
|  | Social Sciences, Law, Arts and Humanities (57.69%) | Engineering and Architecture (43.48%) | Engineering and Architecture (0.00%) |
| **Working conditions in the organization of the destination country** | Engineering and architecture (71.08%) | Sciences  (45.20%) | Health Sciences (25.00%) |
|  | Social Sciences, Law, Arts and Humanities (59.61%) | Social Sciences, Law, Arts and Humanities (30.00%) | Engineering and Architecture (0.00%) |
| **Fringe benefits in the organization of the destination country** | Engineering and Architecture (54.22%) | Sciences  (24.04%) | Sciences  (30.55%) |
|  | Health Sciences (46.94%) | Engineering and Architecture (14.40%) | Engineering and Architecture (0.00%) |
| **Social responsibility in the organization of the departure country** | Health Sciences (74.14%) | Social Sciences, Law, Arts and Humanities (60.00%) | Social Sciences, Law, Arts and Humanities (42.86%) |
|  | Social Sciences, Law, Arts and Humanities (63.46%) | Engineering and Architecture (34.78%) | Engineering and Architecture (12.50%) |
